# The Book World of Medicine and Science

**Published:** 1893-09-02

**Authors:** 


### THE BOOK WORLD OF MEDICINE AND SCIENCE.

### BOOK REVIEWS.

"The School Calendar and Handbook of Examinations, Scholarships, and Exhibitions, 1893," with a preface by F. STORR, B.A., Chief Modern Master, Merchant Taylors' School. Seventh year of issue. (London: Whittaker and Co., 2, White Hart Street, &c.)

The seventh annual edition of the school calendar comes before us this year with valuable additions. By this time we speak of the calendar as an old and tried friend which has filled a real void long felt in the world of tuition. The clear and concise gathering together of all necessary information respecting the centres of learning throughout the British Isles makes this book invaluable not alone to the heads of large and well-known institutions, but to every tutor, guardian, and parent who wishes to place his charges in circumstances most favourable to their special talents; indeed, the question of educating the young should interest all, for it touches upon schemes far wider and more lasting than can be embraced in the training of any individual unit of mankind. In his very able preface to the present edition, Mr. F. Storr sets before us the increasing efforts which are being made to facilitate the education of those whose parents are unable, for want of means, to supply the necessary opportunities for study. The new scheme of open scholarships will bring to the front and train many who would otherwise have been compelled to "hide their light under a bushel." A special section is this year devoted to the review of technical and secondary education. Although the Science and Art Department has done much to promote "manual" instruction, it must be acknowledged that such help as is now hoped for from the ample grant supplied by the Local Taxation Act of 1890, and which fund is now in the hands of the county councils, will materially aid in the organisation of this branch of learning. Reference to this and other parts of the "School Calendar" will be most interesting and instructive to all who give a thought to questions of education, finance, science, art, or even to the consideration of England's imperial relations in future generations; for "men make history." As for the accuracy of the little manual, we have but to look at the compiler's name as a guarantee of its reliability. We have no hesitation in asserting that the public will find in the "School Calendar" much useful knowledge contained in a nutshell.

QUARTERLY JOURNAL OF INEBRIETY.

This periodical is published in Hartford, Connecticut, and the July number, being the third number of the fifteenth volume, has just reached us. It contains several interesting and readable papers, the place of honour being given to Dr. Forel's article on the "Effect of Alcoholic Intoxication Upon the Brain." The subject is treated from the standpoints of evolution and heredity. The evolutionist and the total abstainer will alike rejoice to learn Dr. Forel's opinion "that in the struggle for life, man will never adapt himself to the drinking habit, but that gradually abstinence must triumph, because the drinking world will carry on things more and more extravagant, and will for that be, by the abstainers, outrun." This is very probable; but what about the time necessary to accomplish it? How many generations will have come and

THE BOOK WORLD OF MEDICINE AND SCIENCE—*continued.*

gone before that stage is reached? How many tales of crime and sorrow will have passed before us ere that age has dawned? Dr. Forel is, of course, a thoroughgoing specialist, and we doubt whether the cause he has at heart is likely to be benefited by extreme statements. While we are all agreed that alcohol in anything approaching abuse is greatly to be deprecated, and while we admit that the evil it does far exceeds the good derived from it, we are by no means at one as to the desirableness of expunging alcohol altogether from our dietetic and medicinal tables. The question whether alcohol is or is not food is not likely to be solved by those who begin their investigations with a strong bias on one side or other. Nor can a parallel be fairly drawn between the action of a poisonous dose of alcohol and a dietetic one. It would be as reasonable to condemn opium, "the best drug of all," as a medicine, because men often acquire the opium habit from beginning it as a medicine. No doubt the quantity of alcohol which we would permit would sound absurdly small in the ears of most drinkers; and we believe that Parkes' rule that not more than two ounces of alcohol should be taken during the twenty-four hours errs on the side of excess. But the form in which alcohol is taken counts for something. Drunkenness is rarer among wine-drinking nations than it is with peoples who use any form of malt liquor. Dr. Davis follows with a paper detailing the results of investigations concerning the effects of alcohol on the system. He brings into prominence the conclusions of various experimentalists, but it can hardly be said there is anything strikingly new in the paper, which ends by saying that "alcohol when taken into the system acts directly as an anæsthetic and retarder of all natural metabolism, nutritive, disintegrative, and secretory; and when persistently used causes tissue degenerations that impair health and shorten life." An annotation tells us that of fifteen cases of the drink crave treated in Copenhagen by the gold cure twelve relapsed. Dr. Burson gives us notes of eight cases of delirium tremens treated by hot water. A cupful of hot water was given every hour, and this treatment was followed by such marked success that we should like to see a fair trial made of it in this country. Dr. Crothers, the editor, draws attention to the treatment of kidney mischief by the Turkish bath, and he says that even the gravest lesions of kidneys, liver, and heart are often relieved, sometimes permanently, by baths, diet, and exercise. Unquestionably the Turkish bath as a therapeutic agent is far too little used in England.

PERIODICALS, &c.

The Medical Chronicle has for its chief contents this month articles on Whitehead's operation for hæmorrhoids and tricuspid stenosis, the latter containing notes of several interesting cases, and a copious bibliography. The chief feature of this periodical is its admirable summary of recent work in all departments of medical science selected from the leading British and foreign journals.

The Clinical Journal continues to maintain the supply of excellent clinical lectures for which it is already distinguished. While the most varied subjects are considered, they are always of practical importance, and by writers whose names are well known in connection with the departments of which they treat.

